# Autophagy Ameliorates Reactive Oxygen Species-Induced Platelet Storage Lesions

**DOI:** 10.1155/2022/1898844

**Published:** 2022-04-05

**Authors:** Xi Zhao, Yangchao Zhao, Yanzhong Ding, Yongjuan Ruan, Xiaowei Li, Qi Zhou, Yangfan Zhou, Chunyang Zhang, Liang Hu, Xiaoyan Zhao, Yangyang Liu

**Affiliations:** ^1^Department of Cardiology, Cardiovascular Center, Henan Key Laboratory of Hereditary Cardiovascular Diseases, The First Affiliated Hospital of Zhengzhou University, Zhengzhou, Henan 450052, China; ^2^Department of Extracorporeal Life Support Center, Department of Cardiac Surgery, The First Affiliated Hospital of Zhengzhou University, Zhengzhou, Henan 450052, China; ^3^Department of Cardiology, Hami Central Hospital, Hami, Xinjiang 839000, China; ^4^School of Nursing, Shanghai Jiao Tong University, Shanghai 200030, China; ^5^Department of Thoracic Surgery, The First Affiliated Hospital of Zhengzhou University, Zhengzhou, Henan 450052, China

## Abstract

Platelet transfusion is a life-saving therapy to prevent bleeding; however, the availability of platelets for transfusion is limited by the markedly short shelf life owing to the development of platelet storage lesions (PSLs). The mechanism of PSLs remains obscure. Dissection of the intracellular biological changes in stored platelets may help to reduce PSLs and improve platelet transfusion efficiency. In the present study, we explore the changes of stored platelets at room temperature under constant agitation. We found that platelets during storage showed an increased reactive oxygen species (ROS) generation accompanied with receptor shedding, apoptosis, and diminished platelet aggregation. ROS scavenger reduced platelet shedding but also impaired platelet aggregation. Autophagy is a conserved catabolic process that sequesters protein aggregates and damaged organelles into lysosomes for degradation and platelets' own intact autophagic system. We revealed that there exist a stable autophagic flux in platelets at the early stage of storage, and the autophagic flux in platelets perished after long-term storage. Treatment stored platelets with rapamycin, which stimulates autophagy in eukaryotic cells, markedly ameliorated PSLs, and improved platelet aggregation in response to extracellular stimuli.

## 1. Introduction

Platelets are short-lived (7–10 days) circulating anucleate cells [1], and they play major roles in hemostasis, maintaining vascular integrity, and participate in immune response [2]. Low platelet counts may be achieved by multiple causes, like autoimmune thrombocytopenia, chemotherapy, radiotherapy, genetic disorder, surgery, and trauma caused blood loss, and platelet transfusion is widely used in clinical practices for patients with low platelet counts to reduce bleeding risk. Platelets for transfusion are usually stored in a blood bank at room temperature under constant agitation. However, limited shelf life of transfused platelets (≤5 days) caused seasonal shortages or waste of outdated products. Stored platelets were damaged by deleterious intracellular and extracellular changes (e.g., lactic acid accumulation [3], desialylation [4], PKA-dependent apoptosis [5], and altered ATP generation [6]), which are referred as platelet storage lesions (PSLs). GPIb*α*, GPVI, CD40, and P-selectin are receptors that fall during platelet storage at room temperature [7]. Importantly, GPVI and GPIb-IХ-V are both important receptors involved in platelet activation, and sheddase-mediated loss of platelet receptors reduces the recovery rate and longevity of transfused platelets [7–9].

Reactive oxygen species (ROS) is a variety of oxygen-containing, reactive, and short-lived molecules, such as superoxide anion (O^2-^), hydroxyl radicals (OH^·^), nitric oxide (NO^·^), and hydrogen peroxide (H_2_O_2_) [10]. Inflated cellular ROS generation results in severe damage to DNA, proteins, lipids, and other macromolecules [11]. In addition, ROS may also serve as signal transduction messengers [12]. Recently, multiple studies support the importance of ROS in platelet activation and thrombosis formation [13–15], while imbalanced intracellular ROS during platelet storage has also been reported to be associated with PSLs in both reversible and irreversible ways [16, 17]. Increased ROS in stored platelets may lead to apoptosis [18, 19], P-selectin and CD40 ligand expression, and granule release [20], consistent with the positive regulation of ROS during platelet activation.

Autophagy is one of the major degradation pathways in eukaryotes, characterized by the formation of a double-membrane vesicle to engulf cellular proteins and organelles into lysosomes. Cellular autophagy is constitutively present and could be dramatically induced by environment stress, such as deprivation of nutrients, hypoxia, and compound challenging. Autophagy mediated cellular material recycling, and elimination of damaged organelles sustains intracellular homeostasis [21]. Even though previous studies on autophagy mainly focus on karyotes, Feng et al. reported that autophagy constitutively presents in anucleate platelets, and it can be induced by hunger or rapamycin (a specific inhibitor of mTORC1) [22]. Ouseph et al. confirmed the existence of autophagy flux in resting and activated platelets, and their data also implied that the autophagic flux might be altered in banked human platelets [23]. However, the dynamic change of autophagic flux during platelet storage and the relationship between autophagy and ROS-induced PSLs remain obscure.

In the present study, we revealed that stored platelets showed an increased ROS generation, receptor shedding, and apoptosis. Autophagy is constitutively and inductively present in stored platelets, and there exists a stable but slowly decayed autophagic flux during platelet storage, which finally perished in stored platelets. Manipulating autophagy flux by rapamycin reduces platelet ROS generation and receptor shedding. Moreover, rapamycin improves platelet aggregation in response to extracellular stimuli. The present study on autophagic flux changes during platelets storage may deepen our understanding on the mechanism of PSLs and optimize platelet storage conditions.

## 2. Materials and Methods

### 2.1. Materials

Collagen and ADP for platelet aggregation were from Chrono-Log (Havertown, PA, USA). Rapamycin and bafilomycin were from MCE (Monmouth Junction, NJ, USA). Antibody for LC3 was from Sigma (St. Louis, MO, USA). Antibodies for p62/SQSTM1 and *β*-actin were from Cell Signaling Technology (Beverly, MA, USA). Annexin V-FITC and antibody for human CD42b-FITC, GPVI-PE, CD41-PE, and CD61-FITC were from BD Biosciences (Franklin Lakes, NJ, USA). H2DCF-DA was from Invitrogen™ (Carlsbad, CA, USA). Enhanced chemiluminescence (ECL) for western blotting detection was from Millipore (Burlington, MA, USA).

### 2.2. Platelet Storage Condition

Human PRP (platelet-rich plasma) was collected as previously described [24]. Briefly, whole blood was collected into a 3.8% sodium citrate tube, from blood donors with a written informed consent in accordance with the Declaration of Helsinki, then centrifuged at 150 × g for 20 minutes; PRP were collected into new tubes. All procedures were conducted in a clean bench to avoid contamination. PRP was stored at room temperature in Permalife cell culture bag (OriGen Biomedical, Austin, TX, USA) on a rocking agitator with continuous agitation. Pharmacological intervention (NAC 5 mM, rapamycin 50 nM, or bafilomycin 100 nM) was added before storage.

### 2.3. Experimental Design

**Figure 1:**
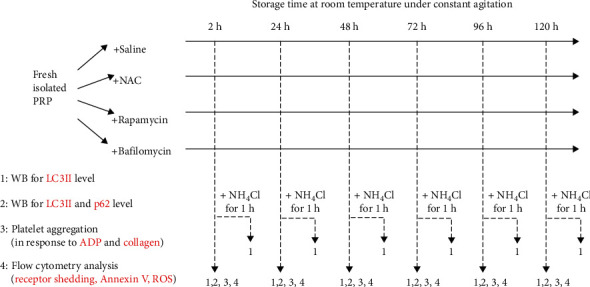
overview for detections of PSLs and autophagy flux during platelet storage.

A flow chart has been present in Figure 1. Briefly, platelets were assigned into 4 groups; vehicle, NAC, rapamycin, or bafilomycin was added into fresh isolated platelet-rich plasma (PRP) before the storage began. For each group at corresponding storage time (2 h, 24 h, 48 h, 72 h, 96 h, and 120 h), PRP is divided into 4 parts for sample collections:

1. PRP (400 *μ*L): PRP is incubated with NH_4_Cl (20 mM) for 1 h; then, washed platelets were isolated and lysed with lysis buffer
2. PRP (400 *μ*L): washed platelets were isolated and lysed with lysis buffer directly
3. 3PRP (≥600 *μ*L): PRP (300 *μ*L) was challenged with ADP or collagen in a Chrono-Log aggregometer
4. PRP (20 *μ*L): PRP was isolated to incubate with designated antibody (anti-GPIb*α*-FITC or GPVI-PE) or fluorochrome (FITC-Annexin V or H_2_DCF-DA) for flow cytometry

### 2.4. Platelet Aggregation

At designated time points (2 h, 24 h, 48 h, and 96 h), PRP was collected from the culture bag. Platelet aggregation was carried out on a 2-channel Model 700 optical aggregation system (Chrono-Log, Havertown, PA, USA), under continuous stirring at 1200 RPM at 37°C. Platelets (300 *μ*L) were stimulated with ADP (20 *μ*M) or collagen (10 *μ*g/mL). Light transmission was recorded for 10 minutes.

### 2.5. Flow Cytometric Analysis of Platelet Receptor Shedding

PRP was collected at designated time points, and platelet concentration was determined with Mindray (BC-2800Vet, Shenzhen, China); platelet GPIb*α* and GPVI surface expression was examined by anti-CD42b-FITC and anti-GPVI-PE. Briefly, 50 *μ*L of PRP (1 × 10^7^/mL) was incubated with 1 *μ*L anti-CD42b-FITC and 1 *μ*L anti-CD41-PE or 1 *μ*L anti-CD61-FITC and 1 *μ*L anti-GPVI-PE for 30 min, then diluted with 450 *μ*L Tyrode buffer. BD Biosciences flow cytometer (Accuri™ C6+) (Franklin Lakes, NJ, USA) and FlowJo V10 software (Treestar, Ashland, OR, USA) were used for analysis.

### 2.6. Measurement of Intracellular ROS

PRP was collected at designated time points, and 10 *μ*L PRP were incubated with 500 *μ*L fluorogenic probe 2′,7′-dichlorofluorescein diacetate (H_2_DCF-DA, 50 *μ*M) for 10 min at 37°C in darkness, then analyzed by flow cytometry immediately. Flow cytometric FCS data analysis was undertaken in FlowJo V10 software.

### 2.7. Annexin V Binding Measurement

Platelet phosphatidylserine expression was evaluated as previously published [24]. Briefly, 50 *μ*L of washed platelets (5 × 10^7^/mL) was incubated with 2 *μ*L of Annexin-V-FITC and 1 *μ*L anti-CD41-PE for 30 min prior to dilution with 500 *μ*L Tyrode buffer containing CaCl_2_ (1 mM). Annexin V binding was immediately assessed on a BD Biosciences flow cytometer (Accuri™ C6+), and FCS data were analyzed with FlowJo V10 software. Platelets were identified by the event characteristic with anti-CD41-PE-positive, forward scatter parameters above 8000.

### 2.8. Immunoblotting Analysis

At designated time points (2 h, 24 h, 48 h, 72 h, 96 h, and 120 h), PRP was collected and stayed at static in the presence or absence of 20 mM NH_4_Cl for 1 hour, then reconstituted with 3-fold ACD buffer, and centrifuged at500 × gfor 10 min. The pellet was lysed with lysis buffer (20 mM Tris and 150 mM NaCl, pH 7.4) containing protease inhibitor, DTT (1.5 *μ*M) and 1 mM PMSF. Loading samples were separated by SDS polyacrylamide gel electrophoresis. The protein was incubated with indicated antibodies overnight. Corresponding secondary antibody incubation was carried out the other day for 1 h at room temperature, with three washes in between incubations with PBS-T buffer. After washing with TBST, proteins were visualized by Tanon 4800 (Tanon, Shanghai, China) after incubation with enhanced chemiluminescence.

### 2.9. Statistical Analysis

All data were analyzed using one-way ANOVA or paired Student's *t*-test to compare normally distributed variables. All data were expressed as mean ± SEM (standard error of the mean), and *P* value ≤ 0.05 was considered as statistically significant.

## 3. Results

### 3.1. Increased Intracellular Reactive Oxygen Species and Platelet Receptor Shedding during Platelet Storage

**Figure 2:**
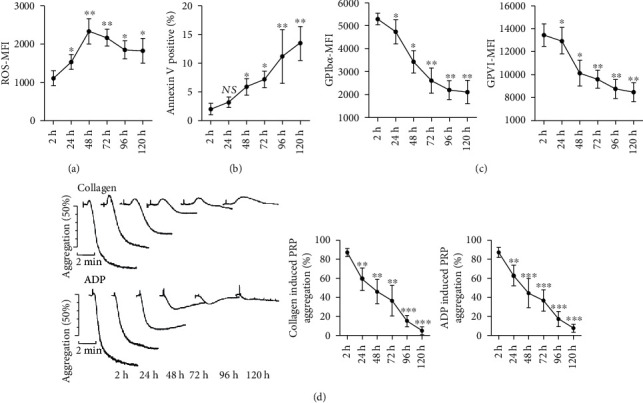
Stored platelets showed increased ROS generation, apoptosis, and platelet receptor shedding accompanied with impaired platelet aggregation. Dot plots illustrate the levels of oxidative stress (a), PS (phosphatidylserine) exposure (b), and platelet receptor expression (GPIb*α* and GPVI) (c) in stored platelets at 2 h, 24 h, 48 h, 72 h, 96 h, and 120 h. (d) Representative aggregation traces and summary of aggregation percentage of stored platelets in response to collagen (10 *μ*g/mL) or ADP (20 *μ*M) after designated storage time (2 h, 24 h, 48 h, 72 h, 96 h, and 120 h). NS: no significance; ^∗^*P* < 0.05; ^∗∗^*P* < 0.01; data were all compared with basal level (2 h) in respective histograms.

To address the oxidative stress changes during platelet storage, we measured ROS generation in stored platelets by flow cytometry using H_2_DCFDA. ROS in platelets increased following the initiation of storage (Figure 2(a)) and slowly decreased at the late stage of storage, implying a diminished intracellular metabolic activity in stored platelets. GPVI and GPIb-IХ-V are important receptors involved in platelet activation, and the loss of platelet receptors attenuates their circulation lifespan and viability, thus impairing the quality of platelet products [16]. Platelets during storage showed a significantly upregulated platelet GPIb*α* and GPVI shedding (Figure 2(b)) and increased apoptosis level by measuring PS exposure (Figure 2(c)), subsequent with reduced platelet aggregation in response to ADP (20 *μ*M) or collagen (10 *μ*g/mL) (Figure 2(d)), which are both important agonists for platelet aggregation and activation.

### 3.2. ROS Scavenger NAC Reduces Platelet Receptor Shedding, Apoptosis, and Aggregation during Platelet Storage

**Figure 3:**
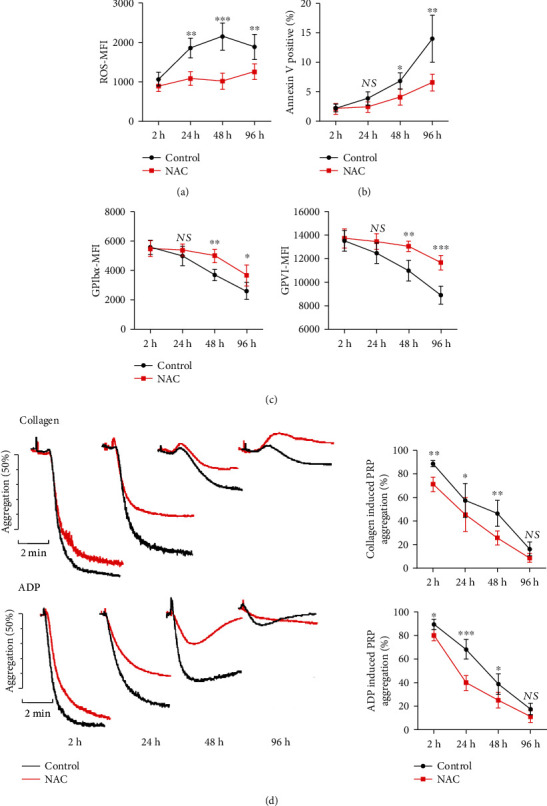
ROS scavenger reduces stored platelet receptor shedding and apoptosis during platelet storage. PRP was pretreated with or without NAC (5 mM), then stored at room temperature on a rocking agitator with continuous agitation. PRP were harvested and analyzed at designated time points (2 h, 24 h, 48 h, and 96 h). Dot plots show the levels of oxidative stress (a), PS exposure (b), and platelet receptor expression (GPIb*α* and GPVI) (c) in stored platelets at 2 h, 24 h, 48 h, and 96 h. (d) Representative aggregation traces and summary of aggregation percentage of stored platelets in response to collagen (10 *μ*g/mL) or ADP (20 *μ*M). NS: no significance; ^∗^*P* < 0.05; ^∗∗^*P* < 0.01.

N-Acetyl-L-cysteine (NAC), a ROS scavenger which acts as a precursor for GSH synthesis [25], potently eliminates ROS generated during platelet storage (Figure 3(a)). To clarify whether ROS scavenger could reduce ROS-induced PSL and prolong platelet activity, we analyze the receptor expression of NAC-pretreated platelets. NAC (5 mM) reduces apoptosis (Figure 3(b)) and GPIb*α* and GPVI shedding (Figure 3(c)). These data indicate that ROS generated during platelet storage plays a critical role in PSL formation. ROS is an important mediator for platelet activation, and the antiplatelet effects of ROS scavenger NAC had been well documented [26]. Consistently, stored platelets showed reduced aggregation activity along with prolonged storage duration (Figure 3(d)), when challenged with ADP (20 *μ*M) or collagen (10 *μ*g/mL).

### 3.3. ROS Scavenger NAC Prolongs the Autophagic Flux in Stored Platelets

**Figure 4:**
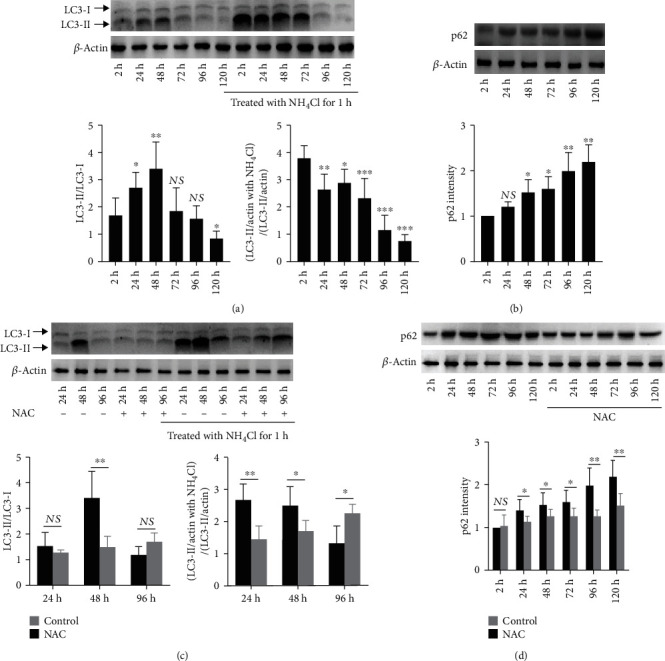
ROS scavenger NAC reduces and prolongs the autophagic flux in stored platelets. PRP was pretreated with or without NAC (5 mM), then stored at room temperature on a rocking agitator with continuous agitation. Harvested PRP were treated with or without NH_4_Cl (20 mM) for 1 h, then centrifuged at 500 × *g* for 10 min and lysed. LC3 levels in stored platelets were analyzed by western blot, and quantification of the ratios of LC3II/LC3I grey intensity was present (a, c). Western blot analysis and quantification of p62 grey intensity of stored platelets were present (b, d). In (a) and (b), summary data were all compared with basal level (2 h). NS: no significance; ^∗^*P* < 0.05; ^∗∗^*P* < 0.01.

We further explored platelet autophagy change during storage by monitoring LC3 (microtubule-associated protein 1 light chain 3) level. During autophagy, LC3-I is converted to LC3-II, which is associated with the phagophore and autophagosome membrane by conjugation to phosphatidylethanolamine, and thus serves as a good autophagy marker. There is a dynamic autophagic flux during platelet storage, and autophagic flux slowly decays after a long duration of storage (Figure 4(a)). However, accumulated LC3-II level in a western blot might also reflect a reduction in autophagosome turnover or the inability of turnover to keep pace with increased autophagosome formation. Therefore, applying LC3-II as an autophagy marker must be complemented by assays to estimate overall autophagic flux to permit a correct interpretation of the results [27]. Stored platelets were collected and treated with NH_4_Cl to block substrate digestion before being subjected to western blot. A more enhanced additive effect of LC3-II levels in the presence of NH_4_Cl indicates a greater induction and cargo sequestration, implying that the storage may enhance autophagic flux through substrate digestion. We monitored autophagy flux intensity in stored platelets by blocking autophagy turnover in the presence NH_4_Cl. After platelets were treated with NH_4_Cl for 1 h, a significantly increased LC3II level was present (*e.g.*, 2 h, 24 h, 48 h, and 72 h) (Figure 4(a)), indicating the presence of an active autophagosome turnover in platelets at the initial stage of storage. However, after 96 h or 120 h of storage, NH_4_Cl treatment does not promote the LC3II level, indicating a perished autophagy flux. Protein SQSTM1, also known as p62, is a selective autophagic adaptor that delivers substrates to autophagosomes and itself is degraded by lysosome along with such substrates [28, 29]. As such, the p62 level is a classical indicator of autophagic flux. It should be noticed that p62 accumulated slowly during platelet storage (Figure 4(b)), which indicates that the basal level of autophagy may be not sufficient to eliminate metabolic waste produced in stored platelets.

We further explore whether ROS scavenger may interfere the active autophagic flux in stored platelets. When treated with ROS scavenger NAC (5 mM) before storage, platelets showed a decreased autophagic flux (Figure 4(c)), and p62 level remains stable during storage (Figure 4(d)), indicating a balanced intracellular autophagosome turnover. To be noted, NAC-treated platelets showed a persisting active autophagic flux at 96 h, as indicated by increased LC3II level when stored platelets were treated with NH_4_Cl for 1 h (Figure 4(c)). These data suggest that ROS scavenger limits the autophagic flux in the early stage of storage and prolongs the existence of active autophagic flux in stored platelets, implying a protective role of autophagic flux on PSLs.

### 3.4. Maintaining Intracellular Autophagic Flux Ameliorates ROS Accumulation in Stored Platelets

**Figure 5:**
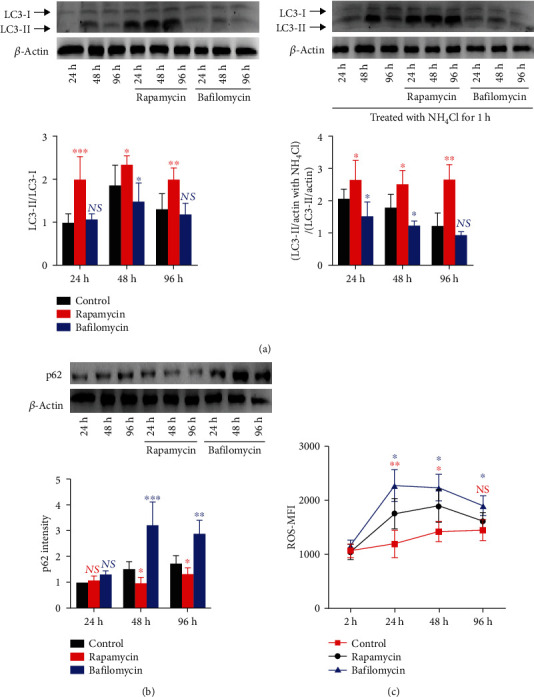
Intracellular autophagic flux maintains lower oxidative stress in stored platelets. PRP was pretreated with rapamycin (50 nM) or bafilomycin (100 nM), then stored at room temperature on a rocking agitator with continuous agitation. (a) Harvested PRP were treated with or without NH_4_Cl (20 mM) for 1 h before being lysed. LC3 levels in stored platelets were analyzed by western blot, and quantification of the ratios of LC3II/LC3I grey intensity (without NH4Cl treated) was present. (b) Western blot analysis and quantification of p62 grey intensity of stored platelets were present. (c) Dot plots illustrate the level of oxidative stress in stored platelets at 2 h, 24 h, 48 h, and 96 h, when treated with rapamycin or bafilomycin. NS: no significance; ^∗^*P* < 0.05; ^∗∗^*P* < 0.01; blue ∗ indicates the comparison between the bafilomycin and control group, while red ∗ indicates the comparison between the rapamycin and control group.

We further analyze whether manipulating autophagic flux might alternately reduce oxidative stress-induced PSLs in stored platelets. Rapamycin is an autophagy inducer by specifically blocking mTORC1 (mammalian target of rapamycin complex 1). Multiple studies showed that low concentration of rapamycin did not influence platelet activation [15, 30]. Rapamycin treatment maintains an enhanced autophagic flux during storage (Figure 5(a)), while bafilomycin, a specific inhibitor of vacuolar H^+^-ATPase, which blocks acidification and protein degradation in autophagosome, significantly abolished platelet autophagic flux (Figure 5(a)). NH_4_Cl incubation more effectively brought up the LC3II level in stored platelet-treated rapamycin than that of control (Figure 5(a)), indicating the existence of active autophagic flux. Furthermore, p62 level remained stable in rapamycin-treated platelets during storage, while p62 level accumulated in stored platelets when treated with bafilomycin, implying an impaired autophagic flux (Figure 5(b)). We also reveal that stored platelets treated with rapamycin showed a lower ROS accumulation (Figure 5(c)), while bafilomycin-treated platelets were accompanied with increased ROS level at the early stage of platelet storage (Figure 5(c)). These data suggest that manipulating autophagy by rapamycin ameliorates ROS accumulation in stored platelets.

### 3.5. Rapamycin Reduces Platelet Storage Lesions and Improves Stored Platelet Aggregation Activity

**Figure 6:**
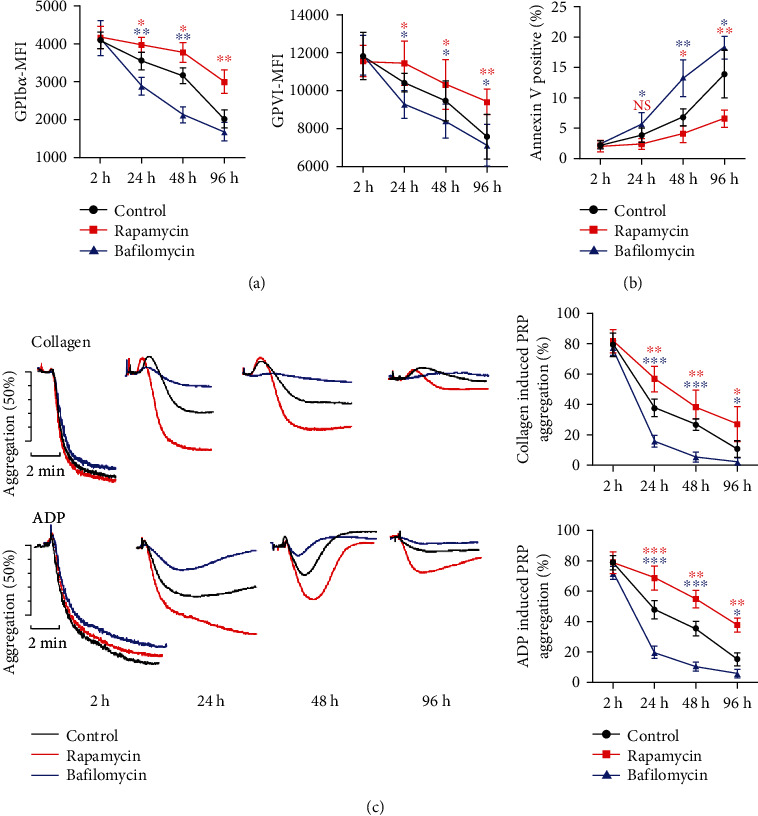
Rapamycin reduces PSLs and improves stored platelet activity. PRP was pretreated with rapamycin (50 nM) or bafilomycin (100 nM), then stored at room temperature on a rocking agitator with continuous agitation. Dot plots illustrate the level of platelets receptor expression (GPIb*α* and GPVI) (a) and PS exposure (b) in stored platelets at 2 h, 24 h, 48 h, and 96 h. (c) Representative aggregation traces and summary of aggregation percentage of stored platelets in response to collagen (10 *μ*g/mL) or ADP (20 *μ*M) after designated storage time (2 h, 24 h, 48 h, and 96 h). NS: no significance; ^∗^*P* < 0.05; ^∗∗^*P* < 0.01. Blue ∗ indicates the comparison between the bafilomycin and control group, while red ∗ indicates the comparison between the rapamycin and control group.

We further explore the effects of manipulating autophagy on ROS-induced PSLs. Platelets were treated with rapamycin or bafilomycin before storage, and our data showed that rapamycin ameliorated platelet GPIb*α* and GPVI shedding (Figure 6(a)), while bafilomycin-treated platelets showed an enhanced receptor shedding (Figure 6(a)). Rapamycin also prevents stored platelets from apoptosis (Figure 6(b)), indicating promoting autophagic flux during platelet storage reduces PSLs. Importantly, we also found stored platelets pretreated with rapamycin showed an improved aggregation activity in response to collagen and ADP, while bafilomycin impaired the reactivity of stored platelets (Figure 6(c)). Taken together, our data suggest that manipulating autophagy flux during platelet storage reduces ROS-induced PSLs and improves platelet reactivity in response to extracellular stimuli.

## 4. Discussion

In this article our data showed that ROS generation during platelet storage is closely associated with PSLs, including apoptosis and receptor shedding. It has been reported that NADPH oxidase (NOX) 1- and 2-derived ROS generation plays important roles in platelet activation [19]; therefore, exogenous ROS scavengers during platelet storage could impair platelet aggregation in response to collagen and ADP, when they effectively reduce PSLs. We also reveal that there is an active autophagic flux during platelet storage, which slowly decays at the late stage of storage, and maintaining a stable autophagy turnover during platelet storage could ameliorate ROS generation and ROS-induced PSLs. Moreover, manipulating autophagic flux by rapamycin during storage dramatically improves platelet aggregation in response to collagen and ADP.

In fact, ROS showed a bidirectional effect on platelet activation, both negative and positive. ROS in platelets may be derived from NOX 1 and NOX 2 activation [26], which are positive regulators during agonist-induced platelet activation. On the other hand, ROS production during platelet storage has been described previously to cause PSLs, such as platelet receptor loss, granule release, and viability impairment [20, 31, 32]. Endogenous ROS produced during platelet storage could be a by-product of respiration chain [19], and accumulated ROS in stored platelets disturbs mitochondrial membrane potential, blocks ATP/ADP exchange translocase at the mitochondrial surface, then subsequently leads to apoptosis [33]. Superfluous ROS result in an increased biomolecule and organelle damage, which consequently promotes intracellular ROS production in a vicious circle [34]. It has been reported that ROS inhibition by NAC or VAS2870 (a specific NOX inhibitor) in stored platelets prevents platelet receptor loss; meanwhile, they do not reduce platelet adhesion and spreading capacity [35]. However, our data showed that ROS scavenger NAC reduced aggregation of stored platelet, indicating that platelet aggregation is a complex process which is sensitive to ROS and ROS-induced PSLs, and reducing oxidative stress during platelet storage is not sufficient to improve platelet aggregation activity.

Baseline autophagy occurs in nearly all eukaryotes to maintain cellular homeostasis and material recycle, and it can be upregulated in response to environment stress including starvation, oxidative stress, and infection [36]. In the present article, we revealed that platelets showed a dynamic autophagic flux during storage process accompanied with increased oxidative stress. We also showed p62 level accumulated in platelets during storage, implying that the basal level of autophagy may not be sufficient to abolish the metabolic waste produced during storage. Upregulated p62 and other autophagy-related proteins in stored platelets [37] might be generated by utilizing mRNAs inherited from megakaryocytes [38], even though platelets are anucleate. Interestingly, high-level R-lipoic acid administration leads to a reduced half-life of circulating platelets, by enhancing autophagy when applied for diabetes treatment [39]. These data indicate that an enhanced regulation of autophagic flux might limit circulating platelet lifespan. This discrepancy might derive from the different concentrations of rapamycin and thus the diffident regulation on autophagic flux. Rapamycin was initially found as an antifungal macrocyclic lactone, then approved for preventing allograft rejection, restenosis, and stent thrombosis [40]. It has been shown that rapamycin induces autophagy by inhibiting mTORC1 signaling, which is a master regulator in the response to nutrient availability and growth factors [41]. Rapamycin is the first reported pharmacological agent to extend the lifespan of mammal and confer protection against aging and age-related diseases [42]. For the concentration of rapamycin, 1000 nM is effective in reducing platelet aggregation [30], while previous studies also showed that 200 nM did not influence platelet aggregation [15]. In fact, 200 nM rapamycin is usually applied to effectively induce autophagy; however, a previous study has reported that apheresis platelets pretreated with rapamycin (200 nM) showed reduced granule release and maximum aggregation in response to ADP and arachidonic acid [37]. Therefore, we applied an uncanonical concentration of rapamycin (50 nM). We speculate that rapamycin (200 nM) is suitable for inducing a sustained autophagy during a short duration, while a long-term and intensive autophagy flux induced by rapamycin (200 nM) may be harmful to platelets, especially when they are anucleate cells.

In the present study, the *in vivo* clearance rate of stored platelets when treated with rapamycin has not been measured, and the lifespan of circulating platelets is partially determined by the glycan modifications of platelet surface receptors [43]. DANA (N-acetylneuraminic acid, 2,3-dehydro-2-deoxy-sodium salt), a neuraminidase inhibitor, has been proved to prevent platelet desialylation and improve platelet posttransfusion recovery [44]. Thus, the effects of rapamycin on the desialylation of stored platelets and the longevity of transfused platelets remain to be elucidated. In addition to glycan modifications of platelet receptors, a further concern is that how autophagy and ROS may regulate GPIb*α* and GPVI shedding, which are known to be predominantly cleaved by a disintegrin and metalloproteinase 10 (ADAM10) and ADAM17 [45]. Previous reports have shown that platelet ADAM17 is activated via a p38 MAPK-dependent pathway, and inhibition of p38 MAPK during platelet storage has been shown to improve the posttransfusion recovery rate [46]. Oxidative stress produced in platelets was rendered as the key regulator of p38 and ADAM17 activation [16]. In addition, enhanced ADAM10 and ADAM17 activity has also been associated with impaired autophagy [47, 48]. Another limitation of the current study is how autophagy may regulate ROS generation during platelet storage. We speculated that increased ROS trends to accumulate misfolded proteins in platelets and subsequently enhances ROS generation. Meanwhile, autophagy could ameliorate ROS-induced intracellular damage and improve energy metabolism efficiency, thus functioning as a protective strategy to degrade ROS-oxidized protein aggregates and altered organelles [49].

To meet the enormous demands of patients in modern medicine, platelet products should be kept adequate in blood banks [50]. Platelet products showed short shelf life and high cost for collection, test, storage, and distribution; thus, they are most affected by fluctuations in practice. A strategy to prolong platelet shelf life would help to minimize platelet product waste and improve usage efficiency. In the study, we have investigated the link between autophagy and PSLs, revealing that rapamycin could partially improve platelet reactivity after storage. Even though the present study focused on the short-term storage and the improvement for storage duration is limited, further investigation on autophagy during storage might help to enhance our understanding on the physiological change of stored platelets and optimize the guidelines for platelet storage.

## Data Availability

The data used to support the findings of this study are available from the corresponding authors upon request.

## Abbreviations

LC3: Microtubule-associated protein 1 light chain 3
mTORC1: The mechanistic target of rapamycin complex 1
NAC: N-Acetyl-L-cysteine
NOX: NADPH oxidase
PRP: Platelet-rich plasma
PS: Phosphatidylserine
PSLs: Platelet storage lesions
ROS: Reactive oxygen species
WB: Western blotting.
